# Supplementary material for: HP1 proteins compact DNA into mechanically and positionally stable phase separated domains
Source: eLife. 2021 Mar 4;10:e64563. doi: 10.7554/eLife.64563 (PMC7932698; doi:10.7554/eLife.64563)
Supplement: Supplementary file 1. [file elife-64563-supp1.docx]

Supplementary Table 1

| HP1α | GKKTKRTADSSSSEDEE**EYVVEKVLDRRVVKGQVEYLLKWKGFSEEHNTWEPEKNLDCPELISEFM**KKYKKMKEGENNKPREKSESNKRKSNFSNSADDIKSKKKREQSNDIAR**GFERGLEPEKIIGATDSCGDLMFLMKWKDTDEADLVLAKEANVKCPQIVIAFYEERLTWHAY**PEDAENKEKETAKS |
| --- | --- |
| HP1β | MGKKQNKKKVEEVLEEEEE**EYVVEKVLDRRVVKGKVEYLLKWKGFSDEDNTWEPEENLDCPDLIAEFL**QSQKTAHETDKSEGGKRKADSDSEDKGEESKPKKKKEESEKPR**GFARGLEPERIIGATDSSGELMFLMKWKNSDEADLVPAKEANVKCPQVVISFYEERLTWHSY**PSEDDDKKDDKN |
| HP1γ | ASNKTTLQKMGKKQNGKSKKVEEAEPE**EFVVEKVLDRRVVNGKVEYFLKWKGFTDADNTWEPEENLDCPELIEAFLNSQ**KAGKEKDGTKRKSLSDSESDDSKSKKKRDAADKPR**GFARGLDPERIIGATDSSGELMFLMKWKDSDEADLVLAKEANMKCPQIVIAFYEERLTWHSC**PEDEAQ |
| HP1α hinge | KKYKKMKEGENNKPREKSESNKRKSNFSNSADDIKSKKKREQSNDIAR |
| HP1α ΔNTE | **EYVVEKVLDRRVVKGQVEYLLKWKGFSEEHNTWEPEKNLDCPELISEFM**KKYKKMKEGENNKPREKSESNKRKSNFSNSADDIKSKKKREQSNDIAR**GFERGLEPEKIIGATDSCGDLMFLMKWKDTDEADLVLAKEANVKCPQIVIAFYEERLTWHAY**PEDAENKEKETAKS |
| HP1α ΔCTE | GKKTKRTADSSSSEDEE**EYVVEKVLDRRVVKGQVEYLLKWKGFSEEHNTWEPEKNLDCPELISEFM**KKYKKMKEGENNKPREKSESNKRKSNFSNSADDIKSKKKREQSNDIAR**GFERGLEPEKIIGATDSCGDLMFLMKWKDTDEADLVLAKEANVKCPQIVIAFYEERLTWHAY** |
| HP1α ΔNTEΔCTE | **EYVVEKVLDRRVVKGQVEYLLKWKGFSEEHNTWEPEKNLDCPELISEFM**KKYKKMKEGENNKPREKSESNKRKSNFSNSADDIKSKKKREQSNDIAR**GFERGLEPEKIIGATDSCGDLMFLMKWKDTDEADLVLAKEANVKCPQIVIAFYEERLTWHAY** |
| HP1α β-hinge | GKKTKRTADSSSSEDEE**EYVVEKVLDRRVVKGQVEYLLKWKGFSEEHNTWEPEKNLDCPELISEFM**QSQKTAHETDKSEGGKRKADSDSEDKGEESKPKKKKEESEKPR**GFERGLEPEKIIGATDSCGDLMFLMKWKDTDEADLVLAKEANVKCPQIVIAFYEERLTWHAY**PEDAENKEKETAKS |
| HP1α γ-hinge | GKKTKRTADSSSSEDEE**EYVVEKVLDRRVVKGQVEYLLKWKGFSEEHNTWEPEKNLDCPELISEFM**KAGKEKDGTKRKSLSDSESDDSKSKKKRDAADKPR**GFERGLEPEKIIGATDSCGDLMFLMKWKDTDEADLVLAKEANVKCPQIVIAFYEERLTWHAY**PEDAENKEKETAKS |
| HP1β α-hinge | MGKKQNKKKVEEVLEEEEE**EYVVEKVLDRRVVKGKVEYLLKWKGFSDEDNTWEPEENLDCPDLIAEFL**KKYKKMKEGENNKPREKSESNKRKSNFSNSADDIKSKKKREQSNDIAR**GFARGLEPERIIGATDSSGELMFLMKWKNSDEADLVPAKEANVKCPQVVISFYEERLTWHSY**PSEDDDKKDDKN |
| HP1γ α-hinge | ASNKTTLQKMGKKQNGKSKKVEEAEPE**EFVVEKVLDRRVVNGKVEYFLKWKGFTDADNTWEPEENLDCPELIEAFLNSQ**KKYKKMKEGENNKPREKSESNKRKSNFSNSADDIKSKKKREQSNDIAR**GFARGLDPERIIGATDSSGELMFLMKWKDSDEADLVLAKEANMKCPQIVIAFYEERLTWHSC**PEDEAQ |
